# Towards Farm Animal Welfare and Sustainability

**DOI:** 10.3390/ani8060081

**Published:** 2018-05-25

**Authors:** Henry Buller, Harry Blokhuis, Per Jensen, Linda Keeling

**Affiliations:** 1 Department of Geography, University of Exeter, Devon EX4 4RJ, UK; 2 Department of Animal Environment and Health, Swedish University of Agricultural Sciences, Box 7068, 750 07 Uppsala, Sweden; Harry.blokhuis@slu.se (H.B.); Linda.Keeling@slu.se (L.K.); 3 Department of Physics, Chemistry and Biology, Linkoping University, SE-581 83 Linkoping, Sweden; perje@ifm.liu.se

**Keywords:** farm animal welfare, sustainability, food security

## Abstract

**Simple Summary:**

The protection and enhancement of farm animal welfare has become an increasingly important component of livestock systems and animal-based food supply chains in many of the more economically developed countries around the world. With the growth of debates around environmental sustainability and food security at the international and global scale, this paper explores the ways in which farm animal welfare, as a public concern, as a science, and as a policy and regulatory area can articulate with these other debates in a comprehensive and holistic manner.

**Abstract:**

As farm animal welfare becomes an increasingly important component of contemporary global livestock production, animal welfare science and animal welfare policy-making need to find new ways of entering global debates over food security and sustainability. In this paper, we explore the means by which both animal welfare science and policy should articulate with these emerging global debates. Having first established the important gains in animal welfare policy and the maturity of animal welfare science, we identify and explore the potential impact of these current debates and argue that they have the potential for profound change in our understanding of, and our response to, the welfare of animals. We conclude the paper with a number of possible recommendations for how a scientifically informed, sustainable animal welfare policy might flourish.

## 1. Introduction

On the 17 October 2016, the United Nations Committee on World Food Security published its ‘Proposed draft recommendations on sustainable agricultural development for food security and nutrition including the role of livestock’. Recommendation ‘D’ of Article VIII, entitled ‘Animal health and welfare’ reads:

‘Improve animal welfare delivering on the five freedoms and related OIE standards and principles, including through capacity building programs, and supporting voluntary actions in the livestock sector to improve animal welfare’.

[1]

The inclusion of this paragraph in the United Nations (UN) document is noteworthy for two important reasons. At one level, the fact that it is there at all is significant. Locating animal welfare within the classic domains of sustainability, namely ‘society’, ‘economics’, and ‘environment’, has always been both complex and problematic. At another level, what is significant is that it has taken so long to get there. Concern about the welfare of kept animals is nothing new. It has been an issue of growing importance to many contemporary societies for over a century, going back to the early British animal protection legislation of the nineteenth century. The non-governmental organization (NGO) World Animal Protection described the UN Recommendation as ‘ground-breaking’ and ‘a massive step forward’ [2] identifying it as the first time in the UN’s 71-year history that animal welfare has been identified as a global goal of sustainable agricultural policy. This Recommendation raises the stakes for animal welfare as a deliverable objective of UN-driven public policy, with all that it implies in terms of programs of investment, technology, expertise, action, education and training, evaluation, transparency and so on. Moreover, it formally identifies animal welfare as a distinct (rather than subsumed) component of sustainable agricultural and economic development, of food security and of human nutrition. Aimed at policy makers, there are echoes here of the earliest UN actions on environmental protection, such as Resolution 2581(XXEV) of December 1969, which set the stage for the 1972 Conference on the Human Environment or the recommendations that emerged from that Conference. If 1972 represented the ‘beginning of modern environmental diplomacy’ [3], might this draft Recommendation not mark a similar nascent stage in international animal welfare diplomacy and if so, how should the animal welfare science and policy communities respond? Although the early animal welfare movement and the early environmental movement share a singular yet foundational moment of collusion in the 1964 publication of Ruth Harrison’s book *Animal Machines* [4] with its forward by Rachel Carson, a major difference between environmentalism in the late 1960s and animal welfare today is that the former had yet to develop its scientific base, its regulatory instruments, and its social and political legitimacy while the latter, over a forty year history, has become a well-established field both in science and policy.

Today, protecting the welfare of farmed animals has unequivocally entered the public policy mainstream in a growing number of countries, with significant public and private regulation governing the welfare of animals in our care. Though, it is worth remembering that formal animal welfare legislation remains largely absent in many countries, including paradoxically in some of the largest animal producing nations of the world (such as Brazil, India, and China). Animal welfare science has, in turn become a well-established discipline in its own right, greatly extending our understanding of positive as well as negative animal physiological and psychological states and the means to appropriately respond to them within the practices of human/animal interaction. The result of this combination of advocacy, science, and policy engagement is that many contemporary livestock farming systems, and other activities in which animals are kept, increasingly operate with a frame of animal welfare rules and concerns. These are driven by an assembly of public legislation, private regulation, market positioning, and individual care, underscored by scientific parameters and methodologies for determining, assessing, and enhancing the welfare of animals.

The growing maturity and embeddedness of animal welfare policy and science, however, brings new challenges, particularly in the context of the increasingly global agendas of food security, climate change, and human diet, along with the practical and ethical questions they raise. Such challenges have potentially major impacts on the future trajectory of welfare science and policy and thereby necessitate informed debate. Two challenges in particular have drawn our attention. The first is the growing incorporation of animal welfare into contemporary understandings of sustainability, now formally endorsed in the United Nations Committee on World Food Security Draft Recommendation. The second is the growing association of animal and human health, increasingly represented by the ‘One Health’ and ‘One Welfare’ agendas [5,6]. Though very different in origin and purpose, we argue that these two challenges, along with other emerging tensions in the positioning of animal welfare science and policy, alter the existing framework for animal welfare science and policy that has developed over the last thirty or forty years and in doing so, offer significant potential, but also concerns, for both. Drawing in part upon the parallel experience of environmental science and policy’s institutionalization over the same period, this paper considers the possible impact of these and other contemporary issues on the forward trajectory of animal welfare science and policy. Having first established the important gains in animal welfare policy and the maturity of animal welfare science, we identify and explore the potential impact of these current debates and argue that they have the potential for profound epistemological, methodological, and even ontological change in our understanding of, and our response to, the welfare of animals. We conclude the paper with a number of possible recommendations for how a scientifically informed, sustainable animal welfare policy might flourish.

## 2. Welfare Gains

In a book written in 2008, Webster [7] set out a series of ‘stepping stones’ for animal welfare science and policy:

1. A clear definition of animal welfare (‘fit and happy’) and a systematic approach to its evaluation;
2. A sound ethical framework that affords proper respect for the value of animals within the broader context of our duties as citizens to the welfare of society and the living environment;
3. Comprehensive and robust protocols for assessing animal welfare and the provisions that constitute good husbandry;
4. An honest policy of education that can convert human desire for improved welfare standards into human demand for these things;
5. Realistic practical step-by-step strategies for improving animal welfare within the context of other equally valid aspirations of society.

Each of these are dynamic targets rather than fixed objectives, shifting and responding to new understandings and circumstances. Yet, we might claim with some conviction that considerable progress has been made in the establishment of at least four of these within mainly Western countries and, we might hope, increasingly beyond partly through the work of international organizations like the World Organization for Animal Health (OIE), World Animal Protection, and others. Progressive and far-reaching definitions of animal welfare, the first of Webster’s ‘stepping stones’, now include both an animal’s ability to ‘cope’ [8] and the expression of positive emotional states [9,10]. A considerable volume of national legislation for the protection of kept animals now exists, constituting a wide-ranging ethical and moral framework for public and private actions. Within the European Union, a substantive number of EU animal welfare related Directives, Regulations and Strategies constitute a supra-national governance framework for animal welfare driven by the principled recognition (written into the 2009 Lisbon Treaty’s amendment of the EU Treaty on the Functioning of the European Union) that animals are sentient beings [11]. Significant regulatory gains have been made in a number of areas, including the bans on excessive confinement of laying hens and pregnant sows [12,13]. Moreover, recent years have seen the multiplication of welfare assessment methodologies and protocols, from the generic approaches produced by Welfare Quality [14,15] to the multitude of private assurance and certification schemes operated by NGOs, retailers, and food sector bodies [16]. 

However, it is in a particular interpretation of the fourth of Webster’s stepping-stones that we observe perhaps the most far-reaching, yet largely unforeseen, change. While there has been little if any coherent public education strategy to improve citizen demand for higher welfare, retailers and the food industry have been highly effective in segmenting the market around the issue of welfare and food quality. This has generated specific welfare-focused marketing and publicity campaigns, NGO strategies, ethical sourcing policies, and the inclusion of farm animal welfare into the remits of the food industry’s collective Corporate Social Responsibility [17]. In essence, this has not only created, at least for some consumers, both the desire and the demand for higher welfare products but has also met that demand, as intended, through the growth of higher welfare supply chains thereby creating, of animal welfare, a distinctive commodity value [18]. Whether this is driven by committed ethics, NGO pressure or by sound marketing sense is open to interpretation. Nevertheless, the net result has been both greater consumer awareness of the welfare issue and an increasing proportion of animal production that meets improved welfare standards, many of which sit higher than regulatory minima [16]. In all, therefore, there is a palpable sense here that, at least within the majority of the Member States of the European Union, animal welfare is now firmly and systematically embedded within political, societal and economic sensibilities, attitudes, and behaviors. Animal welfare has ‘arrived’. 

Moreover, animal welfare stands as a coherent and identifiable scientific discipline, extending from farm animals to laboratory, companion, and zoo animals. Almost 20 years ago, the ethologist Marion Dawkins asked, somewhat provocatively: ‘How can there be a scientific study of animal welfare?’. If “welfare” just means “health,” then is animal welfare not simply veterinary science or animal health studies under another name?’ [19]. Demonstrating that, ‘welfare’ does not mean ‘just health’ and that an animal with poor welfare may be physically healthy and productive but still suffer from a wide range of negative psychological states like fear, frustration, and so on, has been a crucial starting point for this emergent discipline. Animal welfare science developed as a peculiar hybrid, an ‘animalists union’ [20] initially drawing in ‘applied’ ethology, animal production science, and preventative veterinary medicine. From veterinary science and animal biology, animal welfare took the physiological and psychological limits of an animal’s ability to cope with its environment, particularly the physical and social environment of intensive, confined farming systems [21]. From ethology, it took an understanding of how animals normally behave, or should behave, or should want to behave, individually and socially in both natural and in artificial farm environments [22,23]. Finally, from ethics, animal welfare science has reflected the constant interrogation of what is, at any one time, to be considered socially, politically, and hence ethically unacceptable or ‘unnecessary’ in our treatment of animals [24]. Welfare science must also be, in de Greef and Bos’ [25] words, ‘socially robust’, for animal welfare not only operates with a context of potentially competing societal drivers and market forces but is fundamentally a relational science. It has not been concerned with the welfare of a wild squirrel or butterfly (though this may change, see below) but with those animals in whose lives and deaths we are implicated and thereby responsible. Animal welfare science allows us to become, to use Haraway’s [26] term, ‘response-able’. That is to say, it provides not only the means to respond but also the means to identify the needs and wants of those that require our response.

The result brings together a curious epistemology and multiple methodologies, where empiricism, engagement and ethics meet in a desire to know what ‘matters’ for the animal and to ensure, as far as is possible, that what matters is met. In both, animal welfare science has, in many ways, been remarkably successful in providing the evidence and the procedures for defining, identifying, and assessing both good and poor welfare, thereby informing and driving significant legislative and regulatory change [27,28,29]. The results are well documented; the gradual removal of practices of restraint and confinement deemed to cause suffering [30], the outlawing of many forms of manipulation and mutilation [31], the growing use of enrichment to both prevent abnormal behavior and enhance positive states in all forms of captivity [32,33], changes to the physical environment of kept animals to enhance welfare [34], more sensitive management procedures, meeting the behavioral needs of animals and so on. That this should take place within the constraints of the economic and societal realities of a continuously expanding and intensifying livestock industry should not mitigate against its achievements. If animal welfare has ‘arrived’, then, so too has animal welfare science, though, as the Farm Animal Welfare Council [35] and others [36,37] point out, there is still a great deal that needs to be achieved both by science and through policy making and implementation. 

In short, animal welfare science has helped to reassess the parameters of human relations to kept animals. It has done so, first, by bringing the very notion of animal welfare into common use and understanding (though debates around its precise definition and measurement continue [38]. Second, it has shifted the concept of welfare away from the simple removal of physical suffering and towards broader enhancement. Third, animal welfare science has, in association with the wider animal welfare policy community, created the context for reaffirming the moral as well as the economic value of kept animal lives and, fourth, it has provided the means for society to set, empirically and normatively, the limits to what is considered acceptable and what is not in our relations with, and treatment of, animals. These are considerable achievements for an emerging science driven inexorably by what has been a global intensification of animal keeping, whether for food or for companionship. The question we ask in this paper is ‘whither welfare?’; where is it heading and how can animal welfare science, along with the policy arenas it informs, extend its relevance. This, we feel, is a timely question as significant new debates and concerns are driving the trajectory of that intensification as well as driving the nature, direction, and sustainability of our relationship to animals. 

## 3. New Challenges

Formal farm animal welfare science has by and large developed out of, and some would say remains still largely limited to, a Western industrial model of intensive, professionalized and technologized agricultural development. At one level, the Recommendation of the UN Committee on World Food Security with which we started this paper brings the field of (farm) animal welfare science and policy into contact with a vastly different array of social, cultural, and developmental contexts. Yet it does so under the inevitably increasing (and increasingly intensive) livestock production at the global scale. 

Pressures to increase efficiency and intensification of animal agriculture are growing at a time when animal welfare science is concurrently improving our understanding of animals’ needs and preferences, and the extent to which their wellbeing could be compromised by management and husbandry practices [39].

As Lawrence [40] anticipated in 2008, such global issues present both threat and opportunity: the former in the ‘risk that welfare may go backwards in policy terms relative to these emerging issues’; the opportunity being for animal welfare science to work within this emerging policy landscape in a positive and holistic manner. In this final section of the paper, we ask how in the future animal welfare science should extend its relevance and respond to the interlinked challenges of food security, socio-economic development, human well-being, and environmental conservation that this represents.

The challenges facing agriculture over the next half-century are formidable; to be less environmentally damaging yet significantly increase food production while maintaining acceptable levels of animal welfare and human health. To date, farm animal welfare science has proved effective at making intensive production systems broadly acceptable to society [41] by exposing and addressing their excessive harms, by demonstrating their limits in animal terms, and by offering the possibility of aspirational ‘higher welfare’ alternatives [42]. Further intensification, and the spread of the intensive model of livestock production would seem to be, at least in the short to medium term, surely inevitable. As Appleby notes [43], the word ‘intensification’ is problematic for those concerned with the welfare of animals. Issues such as the impact of unnaturally large herd or flock sizes on animal social behavior, the effect of permanent housing and zero-grazing on freedom to express natural behavior, biosecurity and disease risks resulting from selective breeding, medicalization and mutilation and shifts in the role of stockmanship have all been variously identified as causes of concern with respect to intensification, even in its more ‘sustainable’ form [44]. Yet, to be socially legitimate, further intensification should also offer opportunities or aspirations for welfare gains and benefits or, at the very least, the maintenance of current levels of welfare, whether through a new professionalism within intensive systems [41,45] or through new models of financial benefit [46]. Animal welfare science cannot simply reject further intensification on welfare grounds [47]. Neither can it offer unequivocal support for a massive shift to lower-intensity organic and agro-ecological systems, with the inevitable consequences of food prices, food availability and food justice. Nor can it argue for the total rejection of animal husbandry, with its explicit denial of the existence of current and future farmed animals. 

If, following the UN recommendation, improved animal welfare as a goal is to become a significant and recognized component of the global drive towards a more sustainable agricultural development, and if animal welfare science is to retain and build upon its robust and objective science base in driving that development, then we suggest five things need to happen:

1. The welfare of farmed animals needs to become a fully integrated component of sustainability;
2. Animal welfare science and policy need to articulate the emergent concerns for the relationship between human and animal health and welfare;
3. The animal welfare community needs appropriate representation within the international governance structures associated with sustainable agricultural development;
4. Robust and comparable standards for the welfare of farmed animals are needed at an international level across different production systems;
5. Animal welfare science needs to continue to be experimental and innovative in responding to new scientific developments and policy challenges.

### 3.1. Integration 

Intensification in livestock farming reveals animal welfare concern as still largely reactive and, because of that, still largely perceived as a constraint or a challenge (in the negative sense) to human economic activity. There are clear parallels here with the ‘environment’ as a concern in the 1970s, where ‘end-of-pipe’ and ‘low-hanging fruit’ interventions were the default mechanism for environmental protection yet arguably failed to bring substantive and long-term change. In a review, the NGO Compassion in World Farming [48] identified the need for welfare science research to identify how welfare levels can be maintained or improved while meeting food security demands. The findings and practical implications of animal welfare science need to be systematically integrated into sustainable technologies, not as a form of welfare proofing or additional cost but at the stages of both design and implementation of production systems and new technologies, following the model of ‘reflexive’ or ‘ecological modernization’. For Hotzel [49] animal welfare science should be revolutionizing production systems for, as Wathes et al. [47] argue, no system can be considered as ‘sustainable’ if it does not deliver high levels of animal welfare [50]. This is already happening through a growing attention being paid to ‘reflexive interactive design’ and its application, notably to the development of sustainable dairy systems [51,52] and the technological innovations and improved welfare possibilities associated with precision-farming [53,54]. It is happening with the expanding incorporation of welfare criteria in environmental certification and assurance schemes, but these are often limited to relatively high-end food supply chains [55,56]. It is also happening in some of the more innovative responses to legislative change, for example in schemes to enable the enrichment of laying hens following the banning of beak-trimming in the EU [57] and in the emergence of free farrowing systems for pigs following the EU ban on farrowing crates [58].

### 3.2. Articulation

Animal welfare science and policy need to be actively involved with the new agendas of sustainable intensification and food security. To a degree, the UN draft recommendation gives the animal welfare ‘community’ a place at the table, alongside environmental and socio-economic development interests, that it has not enjoyed before. A potentially more significant articulation is however developing in parallel under the emergent banners of ‘One Health’ and ‘One Welfare’. Prompted in part by the growing virulence of zoonotic transmissions and, in part, by a concern for the impact on human health of medicines used in animal farming, the One Health agenda has been referred to as a paradigm shift in human-animal medical interdisciplinarity, though others [59] point out that such collaborations between veterinarians and doctors over issues of public health go back a long way. Arguing strongly for the interdependence of human, animal, and environmental health, ‘One Health’ seeks both visionary change (in acknowledging that interdependence) and practical solutions through combined veterinary, medical, and environmental actions. ‘One Welfare’, a more recent addition to this holistic nomenclature [6] makes similar claims for the interdependence and common consideration of the well-being and welfare of humans and animals and the broader welfare environment [60]. The growing use of these terms and their undoubted policy relevance when we start to think about zoonotic disease, about the often close socio-economic ties in many countries that bind human and animal communities together, or about the impact of human activity (including livestock farming) on the health and welfare of wild animals [61], raises the profile of animal welfare, offers a conduit of responsibility and care, and establishes valuable connections that implicate animal welfare science in new and challenging ways. 

### 3.3. Representation

One notable consequence of the emergence of the ‘environment’ as a global issue of concern in the 1970s was its often innovative institutionalization, notably at the international or transnational level (with, for example, the establishment of the United Nations World Commission on Environment and Development in 1983 and later the UN Conference on Environment and Development in 1992). Within individual countries, the institutional responses have been more variable with distinctive ‘ministries’ of the environment often having a problematic history. More durable than the institutional reconfigurations have been the processes of political modernization [62] where state, market and civil society establish new power relations and regulatory practices around the issue of environmental protection. 

A similar process of political modernization around the issue of animal welfare has recently become visible at the national level in many countries. Examples of this include private regulations and standards superseding public minimum standards, national NGOs and consumer groups setting distinctive welfare agendas, and major retail companies establishing at one and the same time, identifiable areas of corporate social responsibility and a profitable welfare ‘market’ [63]. In other countries, where a tradition of trust in central governmental authorities persists, national regulations and central food chain control remain the norm [64]. 

Yet, at the transnational level, animal welfare is, as yet, relatively poorly represented. The European Union, which has long promoted animal welfare within its own Member States through a variety of policy instruments, has sought to extend the reach of its commitment to animal welfare across an increasingly global field. This it has achieved through trade agreements with third countries, through training initiatives—such as Better Training for Safer Food (BTSF) and its Technical Assistance and Information Exchange instrument TAIEX)—and through its support of the OIE. More recently, the EU Commission has established an Animal Welfare Platform to promote and enhance dialogue on animal welfare and has recently established a series of international animal welfare Reference Centers [65]. The OIE, an intergovernmental body originally established to monitor and control animal disease, has undergone a notable shift in recent years towards the issues and concerns of animal welfare [66,67]. Elsewhere, bilateral initiatives, such as that between the Chinese International Cooperation Committee of Animal Welfare (ICCAW) and the Western NGO Compassion in World Farming have helped create a context for improved welfare through voluntary standards and award schemes. Other calls have emerged for the establishment of an international body through which animal welfare science and research might inform international policy and the transnational governance of animal welfare [68] and the newly established EU Platform mentioned above is clearly a step in this direction. Whether the future holds some animal welfare equivalent to the UN’s ‘Brundland’ Commission on Environment and Sustainability or not, the question of how the global institutionalization of animal welfare science and policy plays out over the next few years will be critical to its success as a viable component of sustainable agricultural development.

### 3.4. Legitimation

One way animal welfare science is legitimated in policy terms has been through the development and establishment of sound criteria for the practical assessment of an animal’s welfare status whether in a farm, a zoo, or the back of a family car. For many years, the aspirational Five Freedoms (freedom from discomfort, hunger and thirst, fear and distress, pain, injury and disease, and freedom to express natural behavior), initially developed by the UK Farm Animal Welfare Council (now Committee) in 1979 have stood as the foundational principles of animal welfare and are widely used as the basis for national regulatory welfare standards in many countries and are specifically referenced in the UN draft recommendation with which we began this paper. More recently, a number of research driven operational assessment schemes have emerged [69,70], while the gradual commodification of animal welfare as a component of market segmentation and brand protection has led to a multiplication of independent private welfare standards in certain countries operated by the food and retail industry and by some animal welfare NGOs. A growing number of these private standards are international in that they regulate distant supply chains for predominantly Western retail chains. Spedding [71] has pointed out that retailers, unlike governments, are not bound by trade rules forbidding the barring of imports on animal welfare grounds.

Nevertheless, there has been, as Horgan and Gavinelli [72,73] note, an absence of international consensus either on the precise role of animal welfare within production systems or on equivalent public standards of animal welfare that might be accepted as universally applicable. The emergence of a global animal welfare agenda, linked both to the regulatory governance of world trade [74] and to sustainable agricultural development, has already led to calls for more systematic and comparable public standards and verification procedures [75]. 

There are three points to be made here. First, as the reach of animal welfare science and policy extends beyond the original context of mitigating the excesses of Western intensification, the Five Freedoms themselves are no longer considered as sufficiently practical to provide a concrete basis for comparative, international welfare assessment [76]. Consequently, a number of alternative and replacement categorizations are emerging from welfare science, including the ‘four principles’ and ‘12 criteria’ of Welfare Quality, the ‘Five Provisions’ or ‘Five Domains’ approach of Mellor and colleagues [77,78] and the ‘Life worth living’ approach [35]. Second, from its position as an intergovernmental organization, the OIE has recently advanced a distinct set of Welfare Standards as part of its Terrestrial and Aquatic Animal Health Codes that are now formally recognized by the UN, alongside the original Five Freedoms in the draft recommendation referred to above. Third, and again following the model of mixed private-public international governance widely, and sometimes controversially adopted for the environmental protection, a specific International Standard Organization (ISO) standard pertaining to animal welfare has recently been established. ISO TS 34700 was formally published in December 2016. Referring directly to the welfare standards of the OIE Codes, it offers a recognized framework for the voluntary adoption of welfare schemes by public and private actors, particularly in those countries where domestic standards and assurance schemes have not been established. Of course, it is too early to assess the impact of these new governance responses but concerns over practices such as ‘greenwashing’, where high impact marketing gives a false impression of actual action, and corporate agenda-setting that occurred under precursor ISOs in the environmental (ISO 14000 and 14001) and quality (ISO 9000) fields [79,80] have provided experience that will be valuable at the time of the three-year assessment of the animal welfare ISO in 2020.

### 3.5. Innovation

Finally, we argue that animal welfare science needs to be experimental and to be open to creative interdisciplinarity to advance knowledge on how animals experience their own situation. It also needs to be innovative in seeking agile responses to the need to achieve durable welfare gains for animals at the same time as promoting lasting socio-economic development and well-being for human communities, particularly in regions threatened by poor food security and poor nutrition. Animal welfare science—and farm animal welfare science in particular—would undoubtedly benefit from greater relative freedom from the restrictive and often reactive research agendas set by the livestock and food industries within which it currently operates. Similarly, the practical testing of measures, procedures, and equipment (including housing systems) to achieve higher levels of animal welfare along with the conversion of existing production systems to higher welfare alternatives would benefit from temporary protection from the rigors of market forces such as has long been afforded organic systems under the EU agri-environmental regime. Although within the EU, financial support under Pillar II of the CAP has been established for the development of animal welfare schemes, and some welfare considerations have been written into EU cross compliance rules, there is little empirical evidence of their effectiveness in maintaining or raising welfare levels. Similarly, as long as animal welfare concern is a mechanism for profitable market segmentation, there will be producers unable to find markets and consumers unable to afford higher welfare products. While private welfare standards and targets such as those operated by the food sector have indubitably helped to raise welfare standards across the board [81], these have also been sometimes socially divisive, particularly within the context of global production chains creating, in some countries, a clear division between high-end high welfare systems for export and low value, low welfare systems for domestic consumption [82]. Interdisciplinary and transdisciplinary research is needed into the mechanisms for achieving improved welfare standards within very different social and economic contexts, for example by utilizing the possibilities of cheaper labor costs to allow for higher levels of stockmanship [83]. The challenge raised by ‘One Health’ and ‘One Welfare’, and by the UN Recommendation is therefore not just between the medical and animal sciences but between these and the environmental, social, and developmental sciences. One clear example of this has been the work funded by bodies like the Brooke and the UK Donkey Sanctuary on how the care of working equids can be better integrated into local developmental objectives within both urban and rural communities in Africa and India [84,85,86]. It is interesting that the welfare of equids (which combine a variety of roles, both economic and social) seems to be the particular area where these sorts of associations are being made. Building on these examples, there is an opportunity here for farm welfare science to become more widely integrated into developmental agendas and the United Nation’s Sustainability Goals in a way that the UN Recommendation clearly anticipates animal farming, especially in those developing countries where agricultural intensification and medicine use are a major concern. 

## 4. Conclusions

Animals are kept for a wide range of human purposes. At a simplistic level, the quality of their lives affects us, not in some Kantian sense of exercising our virtue and moral worth, but in the more prosaic sense of good food, healthy eating, and good companionship. We have argued here for new endeavors and new disciplinary associations around animal welfare. Animal welfare science, as we have seen above, draws upon an increasing range of disciplinary and epistemological traditions and has occasionally turned to philosophy, and particularly ethical philosophy both to locate itself within the context of human-animal relations and in acknowledgement of the ethical complexities of defining the ‘acceptable’ and ‘unnecessary’ animal suffering that are often part of those relations. Its relationship with social science, on the one hand, and environmental studies and ecology on the other hand, has been markedly less sure. A new more holistic approach, grounded in a continuingly robust and objective welfare science, needs to embrace a wider constituency of positions in the face of climate change, food security and human health. This is not to speak less forcibly or less scientifically for the animal, but rather to attain a new relevance and reach in the future. Conceptualizations of animal welfare, both popular and sometimes scientific, often seem to embody a certain nostalgia for a gentler, less modern age (cf. Webster’s ‘Eden’ mentioned above). Certainly, Harrison’s famous 1964 ‘*Animal Machines*’ castigated ‘factory farming’ almost as much for its impact upon the rural landscape and the family diet as upon the welfare of animals concerned. Today’s reality is elsewhere. Over and above the base-level of legal compliance, farm animal welfare has quickly been enrolled as a component of quality product differentiation in wealthy nations. In the face of these new global agendas, the task of a broader animal welfare community is not to provide additional mechanisms for selective market performance but rather to help feed the multispecies world in a healthy and sustainable manner that matters to humans and animals alike.
